# Dual-Band Bent Sensing Textile Antenna Under Dual-Mode Resonance

**DOI:** 10.3390/s25247511

**Published:** 2025-12-10

**Authors:** Zi-Qiang Liu, Nuo Chen, Ke Ma, Yu-Cheng Luo, Xiao-Hui Mao, Jia-Chen Qi, Xiao-Hui Li, Wen-Jun Lu

**Affiliations:** Jiangsu Key Laboratory of Wireless Communications and Internet-of-Things, Nanjing University of Posts and Telecommunications, Nanjing 210003, China; 1023173004@njupt.edu.cn (Z.-Q.L.); 1223066326@njupt.edu.cn (N.C.); 1022173306@njupt.edu.cn (K.M.); 1222067013@njupt.edu.cn (Y.-C.L.); 2021010101@njupt.edu.cn (X.-H.M.); novaqi@foxmail.com (J.-C.Q.); lixh@njupt.edu.cn (X.-H.L.)

**Keywords:** dual-band, microstrip patch antenna, bent sensing, textile antenna, dual-mode resonance

## Abstract

This article presents the design of a dual-mode resonant, dual-band textile microstrip patch antenna for bent sensing applications. The antenna has a simple, slit-perturbed circular sector patch configuration. Unlike traditional single-mode resonant bending sensor antennas, dual-mode resonance brings a unique dual-band sensing characteristic to textile antennas. It effectively covers 2.45 GHz and 5.8 GHz Industrial, Scientific and Medical (ISM) frequency bands. Experimental results demonstrate that the proposed antenna achieves −10 dB impedance bandwidths of 1.4% (2.43–2.465 GHz) and 2.4% (5.775–5.915 GHz), with maximum peak gains of 8.8 dBi and 9.1 dBi, respectively. As experimentally validated on flannel substrates, the antenna achieves maximum bent sensing sensitivities of 1.1 MHz/mm and 1.78 MHz/mm at 2.45 GHz and 5.8 GHz bands, respectively. Furthermore, the antenna is able to provide stable E-plane broadside radiation patterns in bending situations. It would be an ideal candidate for radio frequency identification (RFID), health monitoring systems, and flexible communication applications.

## 1. Introduction

With the rapid development of Internet of Things (IoT) technology, sensing antennas for wearable electronic devices have been attracting increasing attention [1,2]. In particular, the application of deformation sensing holds great potential in the field of wireless body area networks (WBANs) [3] because of its ability to efficiently capture and monitor mechanical quantities such as strain, pressure, and bending [4]. Existing designs of traditional antennas have limited sensing capabilities and lack flexibility, which may make it difficult to address challenges in complex sensing environments [5,6]. This inspires the exploration and development of highly sensitive, multi-functional flexible sensor antennas for intelligent IoT applications.

Microstrip patch antennas (MPAs) have been widely used as conformal sensing antennas owing to their flexibility, robustness, and compact size [7]. One of the representative examples is the MXene flexible MPA [8] for multi-sensing pressure and liquid level applications. Similarly, the MXene MPA on polydimethylsiloxane (PDMS) substrates for strain sensing is developed to enable the effective monitoring of various human motion states [9]. In addition to the aforementioned detections of mechanical quantities through structural deformation, sensing MPAs have also been extended to monitor environmental parameters such as temperature [10], humidity [11], and liquid concentration [12]. Most mechanical bent sensing MPAs are classical rectangular [13,14], square [15], and circular [16] structures. Respectively, they resonate at the fundamental TM_10_/TM_01_ (rectangular/square) and TM_11_ (circular) modes. Square and circular MPAs with a single degree of freedom (i.e., length for square, radius for circular) struggle to operate at multiple frequency bands. Although studies have demonstrated the feasibility of dual-mode resonance (i.e., TM_10_/TM_01_ modes) in rectangular MPAs, such as dual-band temperature sensors [17] and dual-band strain sensors [18], these approaches suffer from a narrow dual-band ratio smaller than 2:1, making them unable to meet wide, cross-band application requirements; moreover, the orthogonal modes at different bands may lead to significant variations in sensing performance. Furthermore, to enhance the sensing performance of the fixed, fundamental mode resonant square and circular MPAs, relatively expensive substrate material [19] or high fabrication complexity [20] are required. This inspires the study on bent sensing MPAs based on distinctive resonant mode families. Recently, the single-mode resonant, circular sector MPA has been validated as a simple bent sensing antenna with two degrees of freedom in design (i.e., radius and central angle) [21]. Inspired by the promising work in inexpensive batch fabrication, we seek to develop a cost-effective, dual-mode resonant bent sensing circular sector MPA with a large dual-band ratio and stable sensing performance.

In this article, a dual-mode resonant, dual-band textile microstrip patch antenna for bent sensing is proposed, which works at the 2.45 GHz and 5.8 GHz Industrial, Scientific, and Medical (ISM) bands. The antenna consists of a circular sector patch loaded by a pair of rectangular slits and an inset microstrip feedline, and employs flannel fabric as the substrate. Its structure is simple and easy to manufacture. Firstly, the dual-mode resonant antenna process is presented. The size of the antenna’s radiation patch is optimized using HFSS simulation software to ensure that |S_11_| < −10 dB in both operating frequency bands. Subsequently, the performance variations of the antenna under bending conditions are verified through numerical simulations and experiments. A set of empirical formulas between bending radius and resonant frequency are fitted and deduced to confirm the feasibility of the dual-band bent sensing antenna.

## 2. Design Process of the Antenna

**Figure 1:**
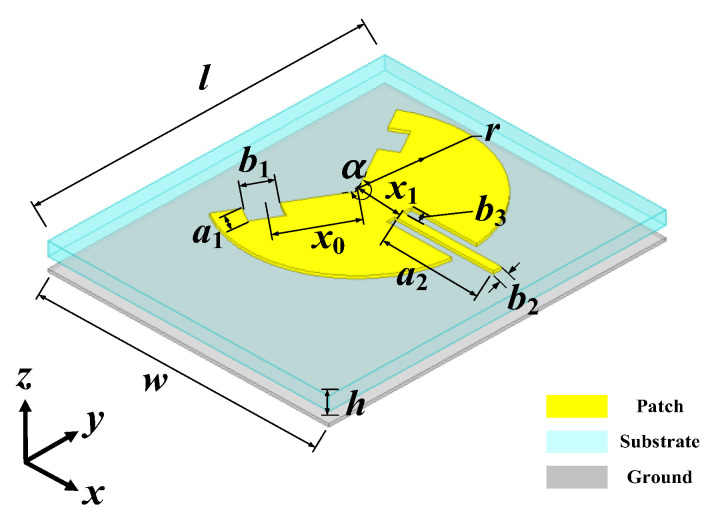
Geometry of the dual-band bent sensing antenna (thickness of ground plane: 0.15 mm).

**Figure 2:**
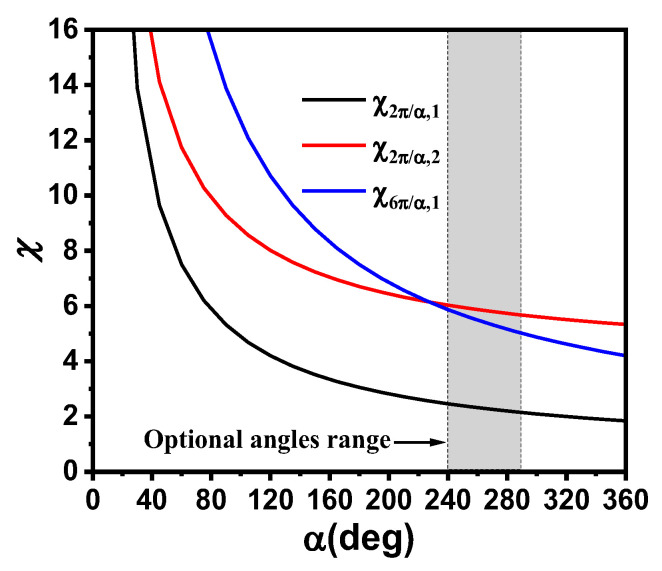
Mode synthesis computation curves of the antenna.

**Figure 3:**
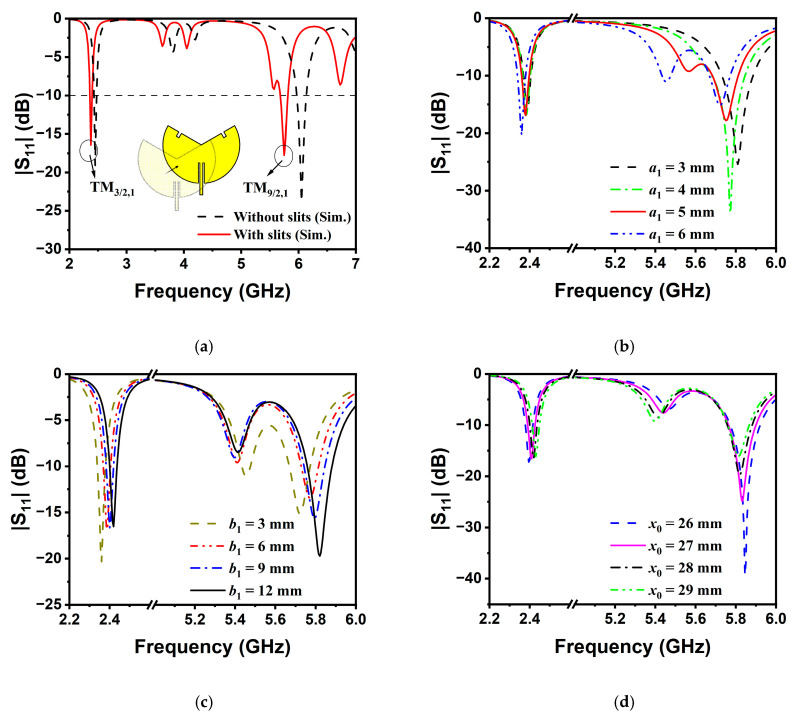
Parametric studies on slit slots. (**a**) Comparison between slit-loaded (*a*_1_ = 5 mm, *b*_1_ = 3 mm, *x*_0_ = 27 mm) and slit-unloaded cases; (**b**) *a*_1_ (*b*_1_ = 3 mm, *x*_0_ = 27 mm); (**c**) *b*_1_ (*a*_1_ = 6 mm, *x*_0_ = 27 mm); (**d**) *x*_0_ (*a*_1_ = 6 mm, *b*_1_ = 12 mm).

The geometry of the dual-band bent sensing antenna on a flexible flannel substrate with a thickness of *h* = 0.8 mm and relative permittivity *ε_r_* = 1.3 is illustrated in Figure 1. The geometry of the dual-band bent sensing antenna (thickness of ground plane: 0.15 mm) consists of a circular sector patch antenna with two open-circuited radii fed by an inset microstrip line [22]. Our design aims to cover both the 2.45 GHz and 5.8 GHz ISM bands. According to the analytical results in [23], the central angle α should be carefully selected, which implies the principal excitation of TM_2π/α,1_ mode at the 2.45 GHz band. In order to yield large dual-band ratio operation and ensure coverage of the 5.8 GHz band, the TM_6π/α,1_ mode can be excited for operation. Accordingly, the central angle should be selected within the range of 240° to 290° (i.e., corresponding to the resonance frequency of TM_6π/α,1_ mode between 5.726–5.843 GHz): Figure 2 gives the mode synthesis curves for the dual-mode resonance designs. According to the analytical theory [23], the eigen root of the first higher-order, radially resonant TM_2π/α,2_ mode is nearly equal to that of the TM_6π/α,1_ mode, i.e., χ_2π/α,2_ ≈ χ_6π/α,1_. Therefore, the higher-order TM_2π/α,2_ mode should be detuned outside the frequency range formed by TM_2π/α,1_ and TM_6π/α,1_ modes. As highlighted in Figure 3a, the central angle is determined as α ≥ 240° to detune and suppress the undesired higher-order radially resonant mode. Without loss of generality, *α* is set to 240°. According to (1), the radius *r* is 41.8 mm. The principal TM_3/2,1_ mode resonates at the 2.45 GHz band. A pair of slits is incorporated on the radii to perturb and excite the higher-order TM_9/2,1_ mode resonance at 5.8 GHz. The remaining parameters, including the position *x*_0_, length *b*_1_, and width *a*_1_ of the slits; feed position *x*_1_; and length *a*_2_ of the feedline, are numerically determined using HFSS simulations in the following steps.

(1) $$r\approx \frac{2.463c}{2\pi f_{0}\sqrt{\varepsilon_{r}}}$$

Here, *f*_0_ is the corresponding center frequency, and *c* is the speed of light in vacuum.

Figure 3a illustrates the wideband reflection coefficient frequency responses varying from 2 to 7 GHz for slit-loaded and unloaded antennas. In the initial step, the parameters of the slits are empirically chosen by the higher-order mode function [23,24,25], the dimension (i.e., *a*_1_ × *b*_1_) for the slit slots is chosen to be 5 × 3 mm^2^, and the position is set at *x*_0_ = 27 mm. It can be seen that the TM_9/2,1_ mode has been excited, perturbed, and tuned to the desired 5.8 GHz band by incorporating slits. The center frequencies of the TM_3/2,1_ and TM_9/2,1_ modes are 2.38 GHz and 5.76 GHz, respectively [21,26]. The effects of the most sensitive parameters of *a*_1_, *b*_1_, and *x*_0_ were numerically simulated and are shown in Figure 3b–d. It can be observed that the dual-band ratio is more sensitive to the slits’ length than their width and positions. Finally, the parametric studies yielded the values of all key parameters tabulated in Table 1.

**Figure 4:**
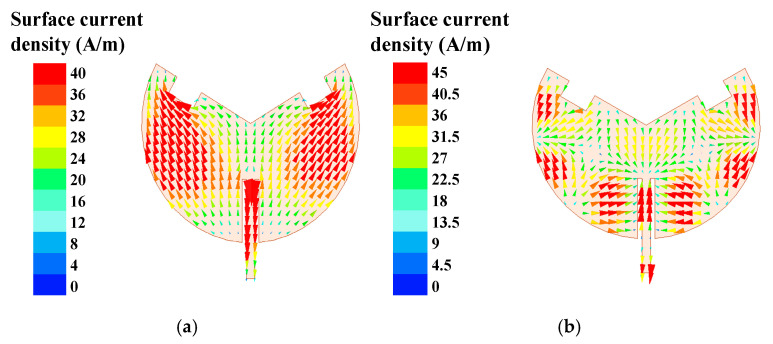
Simulation results for surface current density distributions: (**a**) 2.45 GHz and (**b**) 5.8 GHz.

The surface current density distributions at 2.45 GHz and 5.8 GHz are numerically simulated and presented in Figure 4. As can be seen, the proposed antenna simultaneously operates under TM_3/2,1_ and TM_9/2,1_ dual-mode resonance for radiation, which is consistent with the phenomenon described in [23]. It evidently validates the dual-mode resonance mechanism.

## 3. Investigations on Mechanical Bent Sensing

**Figure 5:**
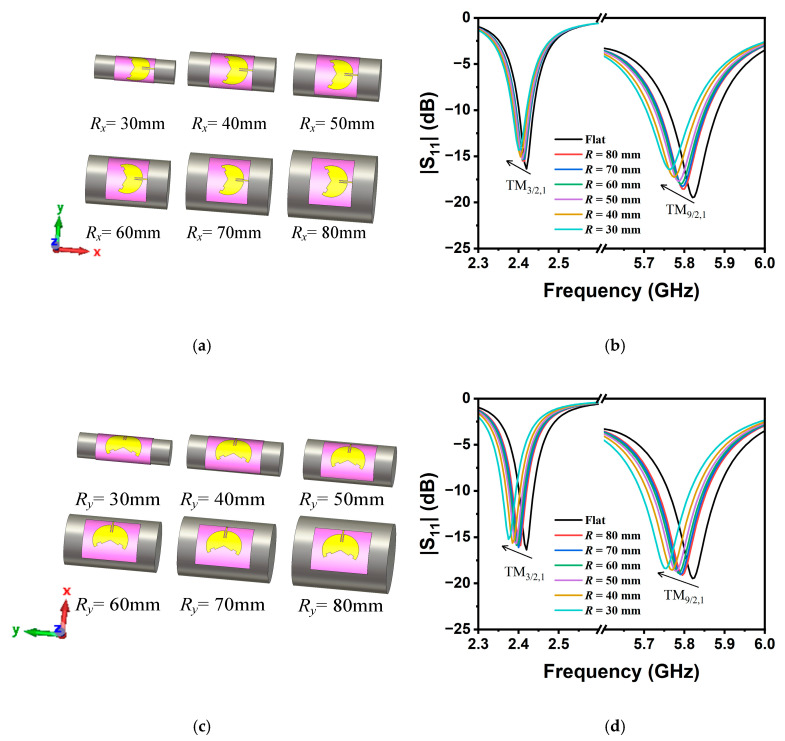
(**a**) The antenna bent at different radii along the *x*-axis; (**b**) simulated S_11_ plot at different bending radii; (**c**) the antenna bent at different radii along the *y*-axis; and (**d**) simulated S_11_ plot at different bending radii.

To evaluate the performance of the antennas for bent sensing, it was necessary to conduct curvature sensitivity testing along different directions. Simulations on mechanical bent sensing were conducted by bending the sensing antenna aligned along the *x*- and *y*-axis directions, respectively. The range of the cylinders varied from 30 mm to 80 mm, with the corresponding situations shown in Figure 5a,c. With the decrease in the curvature radius, the shift of |S_11_| was significant, as illustrated in Figure 5b,d. It is observed that regardless of the bent direction, the center resonant frequencies of both operating modes exhibited consistent trends of change (i.e., frequency shifts toward lower frequencies for smaller *R*). The maximum frequency shifts did not exceed the ranges of both ISM bands. It is recommended that the antenna should demonstrate stable, isotropic, bent sensing characteristics at both bands.

**Figure 6:**
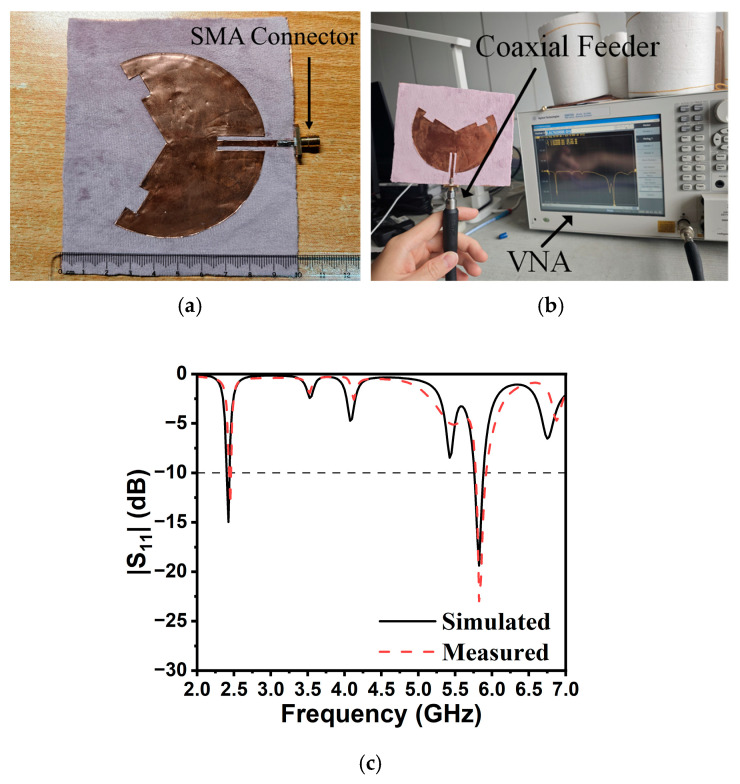
(**a**) Photograph of the fabricated dual-band bent sensing antenna prototype; (**b**) measurement using the E5072A VNA; and (**c**) simulated and measured reflection coefficients.

Based on the numerical simulation results, the experimental validation was carried out. Firstly, a batch of antenna prototypes were fabricated: the patch, ground plane, and feedline were fabricated using 3M’s adhesive copper tape on a flannel textile, as shown in Figure 6a. Subsequently, the antennas were measured using Keysight’s E5072A vector network analyzer (VNA), as shown in Figure 6b. As illustrated in Figure 6c, the measured and simulated results are well matched. The discrepancies of resonant frequency are caused by the unpredicted deviation of relative permittivity and the structural non-uniformity of the flannel textile. The measured impedance bandwidths for |S_11_| < −10 dB were 35 MHz (2.43–2.465 GHz, 1.4%) and 140 MHz (5.775–5.915 GHz, 2.4%), respectively. Evidently, the TM_3/2,1_ and TM_9/2,1_ modes were properly tuned to cover most of the two desired ISM bands (i.e., 2.4–2.484 GHz and 5.725–5.875 GHz).

**Figure 7:**
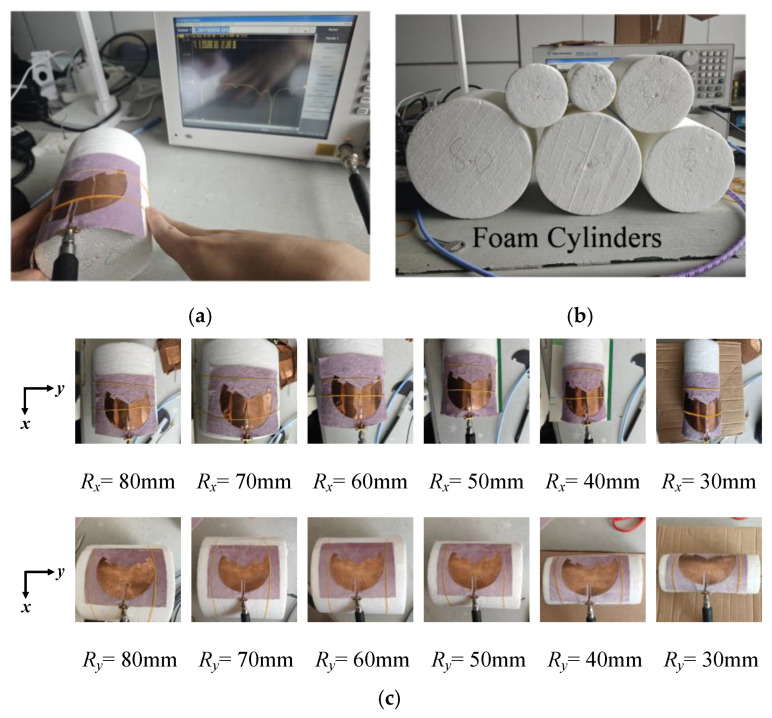
(**a**) Testing of bent sensing; (**b**) foam cylinders of different radii; and (**c**) the antenna is mounted on a foam cylinder and bent along the *x*-axis and *y*-axis.

**Figure 8:**
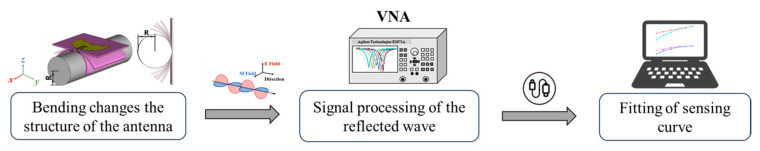
Schematic illustration of antenna bent sensing experiment.

Then, the antenna was tested for bending. In the experiment, the textile antenna was fixed onto the foam cylinder using adhesive tape and rubber bands to emulate the conformal bending condition. The variations of |S_11_| in different bent cases were acquired by the VNA, as illustrated in Figure 7a. To ensure consistency with the simulation conditions, the radius of the foam cylinder was identical to that used in the simulation, as shown in Figure 7b,c. Finally, the measured data were fit and analyzed to yield closed-form sensing sensitivity formulas. A flowchart of the mechanical bent sensing studies is shown in Figure 8.

**Figure 9:**
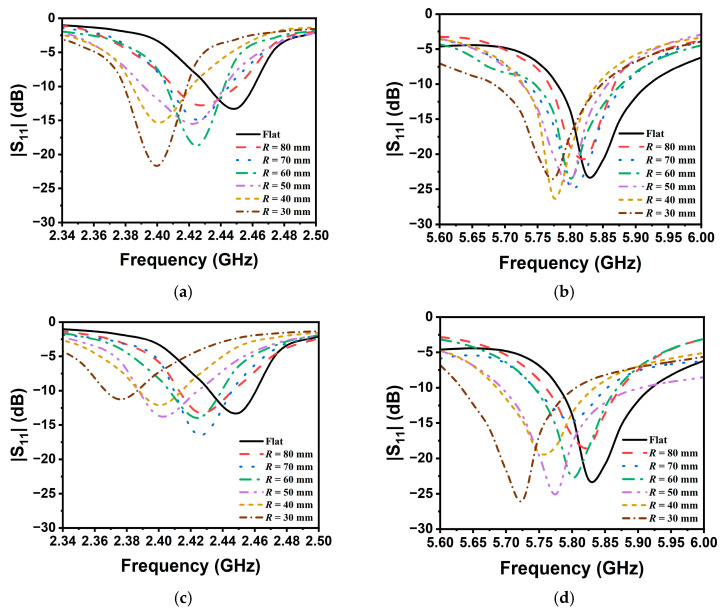
Measured |S_11_| on different bending radii: (**a**), (**b**) along the *x*-axis at 2.45 GHz and 5.8 GHz, respectively; (**c**), (**d**) along the *y*-axis at 2.45 GHz and 5.8 GHz, respectively.

The experimentally measured |S_11_| curve is shown in Figure 9. The trend of the center resonant frequency shift matches the simulation results. It is seen that the frequency variation at 5.8 GHz under the TM_9/2,1_ mode resonance is more sensitive than the TM_3/2,1_ mode resonance at 2.4 GHz. When the bending radius is 30 mm, the maximum frequency shifts of the lower-order and higher-order modes are 75 MHz and 105 MHz, respectively. Moreover, a comparison between Figure 9a–d reveals that bending along the *y*-axis induces larger frequency shifts than the *x*-oriented case. The reason for this is that bending along the *y*-axis (perpendicular to the current direction) can more effectively disturb the structure and surface current path of the antenna, resulting in stronger sensing capability, whereas bending along the *x*-axis (parallel to the current direction) produces a weaker disturbance effect [21]. Excessive frequency shifts may potentially cause the resonant frequency point to fall outside the detectable ISM band. To suppress this effect, bending sensing can be selected along the *x*-axis direction.

**Figure 10:**
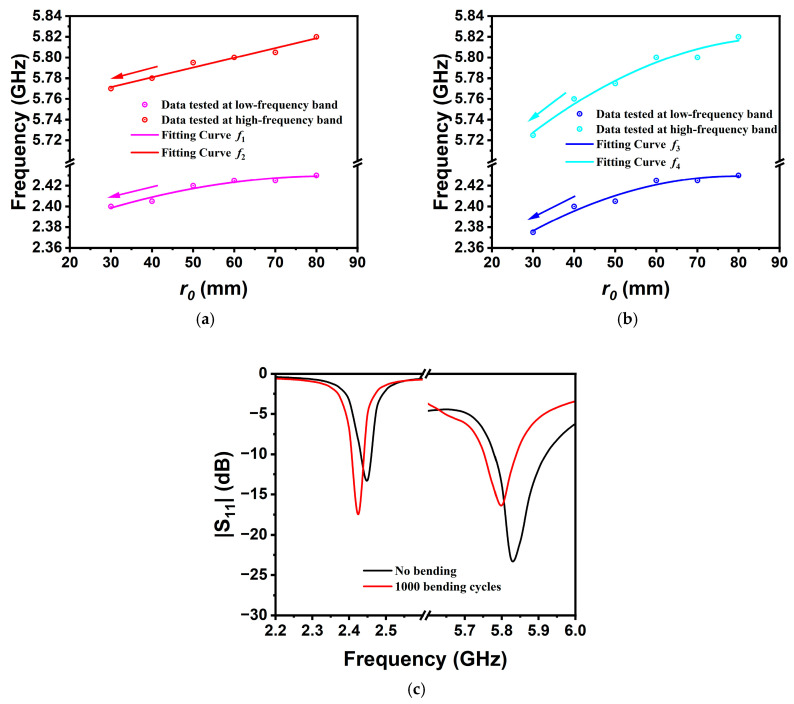
Fitting curves and resonant frequency versus radius (**a**) along the *x*-axis and (**b**) along the *y*-axis, and (**c**) resonant curve after 1000 bending cycles.

To effectively measure its sensitivity, we established a quantitative relationship between the bending radius and the frequency shift, as shown in Figure 10a,b. Correspondingly, it yielded the empirical formulas *f*_1_, *f*_2_, *f*_3_, and *f*_4_ expressed by (2), (3), (4), and (5), where *R*^2^ represents the goodness-of-fit for the functional model, and the unit of *r*_0_ is mm. As can be seen, the frequency shift versus bending radius exhibits a nearly linear relationship, with a relatively small coefficient of the square term.

Fitting curve of the bending sensor along the *x*-axis:

(2) $$\begin{array}{cc} f_{1}(\mathrm{MHz})=2354.43+1.7r_{0}-0.011{r_{0}}^{2} & R^{2}=0.92252 \end{array}$$

(3) $$\begin{array}{cc} f_{2}(\mathrm{MHz})=5743.14+0.943r_{0} & R^{2}=0.9654 \end{array}$$

Fitting curve of the bending sensor along the *y*-axis:

(4) $$\begin{array}{cc} f_{3}(\mathrm{MHz})=2293.29+3.41r_{0}-0.021{r_{0}}^{2} & R^{2}=0.94481 \end{array}$$

(5) $$\begin{array}{cc} f_{4}(\mathrm{MHz})=5616.68+4.42r_{0}-0.024{r_{0}}^{2} & R^{2}=0.95966 \end{array}$$

The sensitivity of the antenna sensor can be expressed mathematically as the ratio of frequency shift Δ*f*_0_ and radius variation Δ*r*_0_.

(6) $$Sensitivity=\frac{\Delta f_{0}}{\Delta r_{0}}\mathrm{MHz}/\mathrm{mm}$$

Calculations show that the maximum sensitivities of the TM_3/2,1_ and TM_9/2,1_ modes are 1.1 MHz/mm and 1.78 MHz/mm, respectively. Table 2 shows the comparative analysis of the proposed prototype antenna and existing bent sensing antennas in terms of manufacturing materials, physical dimensions, operating modes, and sensitivity. The proposed bent sensing antenna not only achieves cost-effective and stable multi-band sensing, but also realizes a large dual-band ratio up to 2.38:1. Figure 10c shows the |S_11_| curve after 1000 bending cycles. It can be seen that the resonant frequencies still fall within the respective 2.45-GHz and 5.8-GHz ISM bands. This validates the robustness, stability, and durability of the antenna design. These advanced characteristics make it highly suitable for smart sensing systems, which is of great significance for promoting the application of antennas in medical health monitoring and human–computer interaction fields.

**Figure 11:**
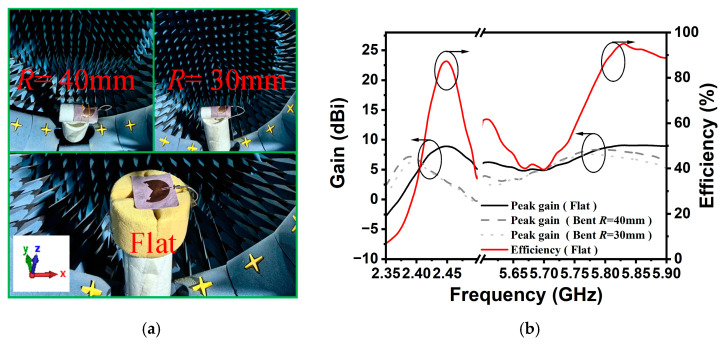
Radiation performance test. (**a**) Far-field test scenarios for sensing antennas, and (**b**) measurement gain and radiation efficiency of sensing antennas (flat and bent).

The radiation characteristics of the bent sensing antenna were measured in a Microwave Vision Group (MVG) Starlab anechoic chamber for both flat and different bending radius states (i.e., *R* = 40 mm and *R* = 30 mm), as shown in Figure 11a. As illustrated in Figure 11b, the measured results indicate that the proposed antenna achieves peak gains of 8.8 dBi and 9.1 dBi at the 2.45 GHz and 5.8 GHz ISM bands, respectively. In the bending situations, the gain at the high-frequency band remains nearly unchanged, while a slight reduction is observed at the low-frequency band. Furthermore, the radiation efficiency of the antenna sensor exceeds 50% across most operating bands when unbent, indicating that the bent sensing antenna meets the requirements for intelligent wearable devices.

**Figure 12:**
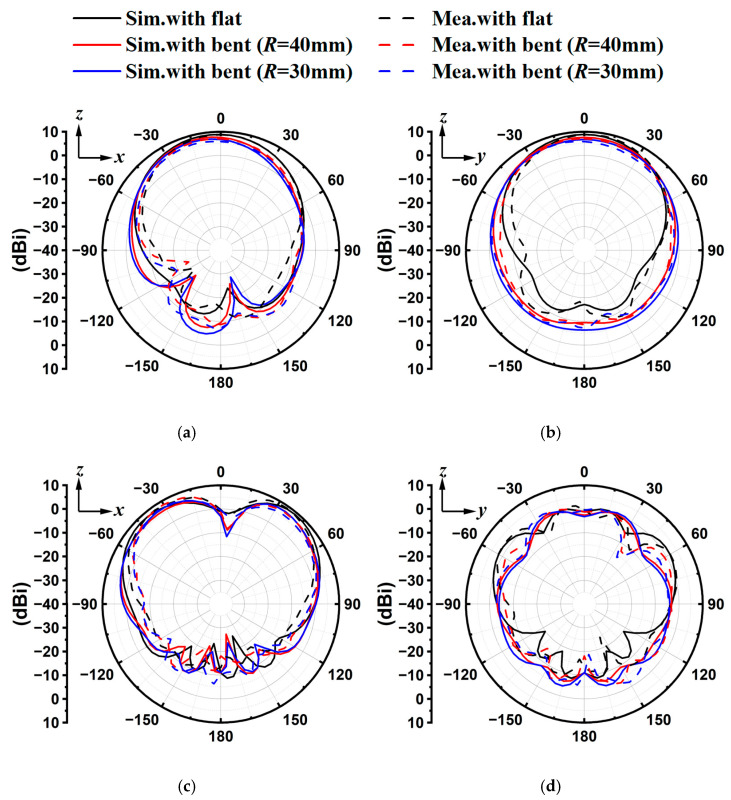
Simulated and measured results of far-field radiation from the *zx*-plane and *zy*-plane for flat and bent antennas at (**a**,**b**) 2.45 GHz and (**c**,**d**) 5.8 GHz.

The radiation patterns in the E-plane (*zx*-plane) and H-plane (*zy*-plane) are plotted in Figure 12. The experimental test results are in good agreement with the theoretical simulation. When the antenna is bent, the main lobe of the radiation pattern is rarely affected, resulting in a slight gain decrement and an increased back lobe level in the low-frequency band. At 5.8 GHz, the radiation pattern of the E-plane exhibits a stable, directional, slightly split beam, with peak gain deviating from the boresight +*z*-direction. Obviously, the dual-band bent sensing antenna exhibits directional radiation characteristics at both bands. Such performance makes it suitable for safety sensors and health monitoring devices in WBANs, since the unidirectional beam may alleviate the reactions between the sensor and human/animal bodies [27]. Hence, the proposed antenna is a promising candidate for wearable devices.

**Figure 13:**
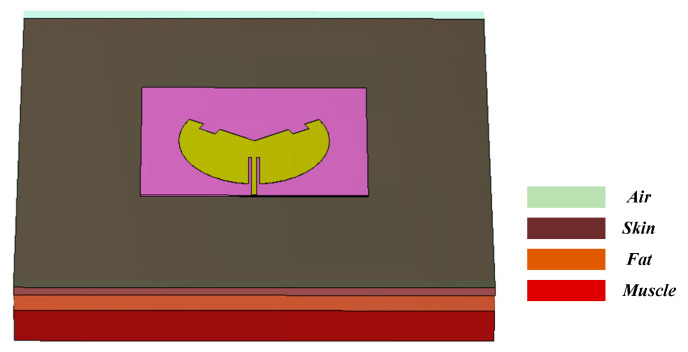
Classical three-layer structure model of human tissue.

For safety considerations, when antennas are used in wearable devices, the Specific Absorption Rate (SAR) is necessary to evaluate the impact of electromagnetic radiation on human health. This prevents radiation from exceeding the human body’s maximum tolerance limits, thereby causing harm to the body. Currently, there are two IEEE standards for measuring SAR values: the SAR limits must not exceed 1.6 W/kg per 1 g of tissue and 2 W/kg per 10 g of tissue [28,29]. In this work, a 250 × 250 × 17 mm^3^ three-layered human tissue section was modeled, as shown in Figure 13. The human tissue model comprises skin of 2 mm thickness, fat of 5 mm thickness, muscle of 10 mm thickness, and a 5 mm air gap between the antenna sensor and the human body. The characteristics of permittivity, conductivity, and mass density [30] for each layer are listed in Table 3. The antenna input power of 100 mW was selected [31]. The human tissue model and the SAR calculations were performed by the electromagnetic simulation software CST 2022.

**Figure 14:**
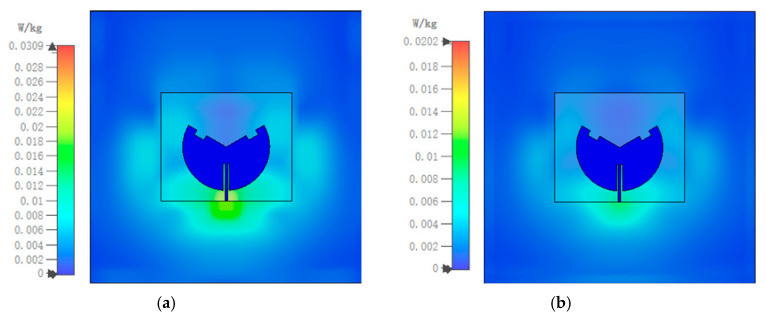
SAR limits: (**a**) 1 g at 2.45 GHz and (**b**) 10 g at 2.45 GHz (with an input power of 100 mW).

**Figure 15:**
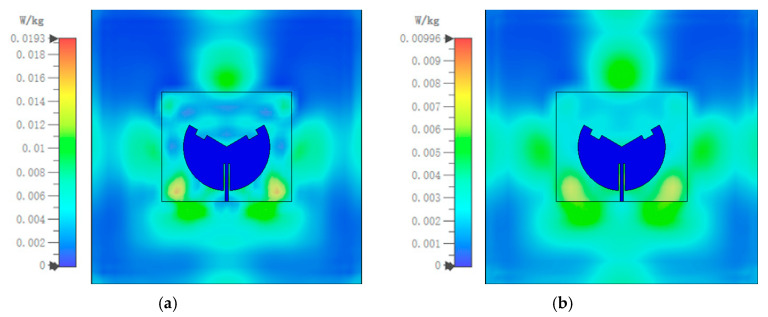
SAR limits: (**a**) 1 g at 5.8 GHz and (**b**) 10 g at 5.8 GHz (with an input power of 100 mW).

**Figure 16:**
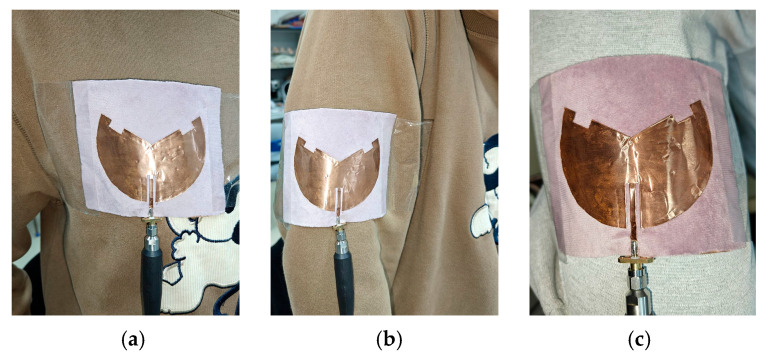
On-body |S_11_|test at different positions: (**a**) chest, (**b**) arm, (**c**) lap.

**Figure 17:**
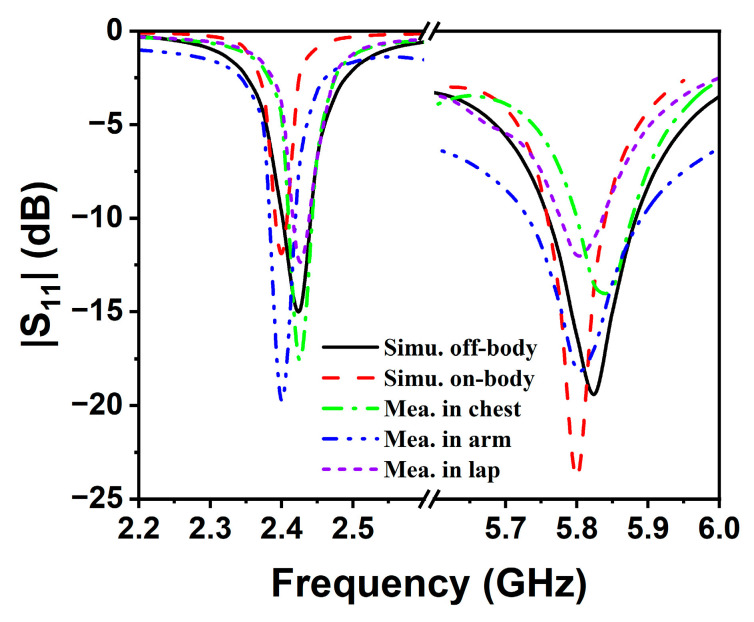
Measured results of |S_11_| with the antenna in free space and on lap and arm.

According to Figure 14a,b, the SAR limits at 2.45 GHz for 1 g and 10 g of human tissue are 0.031 W/kg and 0.02 W/kg, respectively. Furthermore, the SAR limits at 5.8 GHz are 0.019 W/kg and 0.01 W/kg for 1 g and 10 g of human tissue, respectively, as shown in Figure 15a,b. It can be seen that the maximum SAR values at both operating frequency points are much lower than the maximum values specified in the IEEE standard. In addition, Figure 16a–c show the antenna’s reflection coefficient frequency responses when attached to different human positions, i.e., chest, arm, and lap. Owing to its microstrip patch configuration, the mutual coupling with human body is quite minor: the simulated and measured |S_11_| curves are slightly affected. The resonances still fall within the desired 2.45/5.85 GHz ISM band, as shown in Figure 17. Hence, the electromagnetic radiation generated by the proposed antenna sensor is safe for the human body and suitable for wearable applications.

## 4. Conclusions

This article presents a dual-mode resonant, dual-band textile microstrip patch antenna for bent sensing applications. The slit-loaded circular sector patch antenna was fabricated using inexpensive flannel fabric, and a set of bent sensing formulas was deduced. As validated, the overall design features a series of merits, i.e., simple structure, low complexity, and dual ISM-band bent sensing functionality. Compared with existing flexible sensing antennas, the proposed antenna supports higher-order mode resonance sensing functionality while maintaining low fabrication costs. The eigenmode theory [25]-inspired, multimode resonant design methodology is generalized, and it can be extended for implementation with arbitrary inexpensive textiles or flexible substrates. Therefore, the proposed design approach exhibits robustness and cost-effectiveness. It is a promising approach for application in future multi-band sensing and intelligent monitoring systems.

## Figures and Tables

**Table 1:** Design parameters of antenna.

Parameter	Value (mm)	Parameter	Value (mm)
*l*	120	*a*_2_	34
*w*	100	*b*_1_	11
*h*	0.8	*b*_2_	3.3
*α*	240°	*b*_3_	1.5
*r*	41.8	*x*_0_	28
*a*_1_	5.8	*x*_1_	16

**Table 2:** Comparison of the proposed antenna and reported flexible antennas.

Ref.	Material	Dimensions (mm)	Mode	*f*_0_ (GHz) *	Sensitivity
[14]	Graphene and cellulose paper	119.4 × 70 × 0.46	TM_01_	1.63	-
[15]	Graphene and PET	50 × 50 × 0.09	TM_10_	3.62	-
[16]	Copper and FR-4	150 × 150 × 1.5	TM_11_	1.5	-
[21]	Copper and PDMS	100 × 100 × 0.7	TM_4/3,1_	2.45	1.767 MHz/mm
This work	Copper and Flannel	120 × 100 × 0.8	TM_3/2,1_/TM_9/2,1_	2.45/5.83	1.1 MHz/mm, 1.78 MHz/mm

*: *f*_0_ denotes the center frequency without bending.

**Table 3:** Physical parameters of human tissue.

Layer	Permittivity	Conductivity (S/m)	Mass Density (kg/m^3^)
Skin	35.11	3.72	1100
Fat	4.95	0.29	910
Muscle	48	4.96	1040

## Data Availability

The data used to support the findings of this study are available from the corresponding author upon request.
